# Vitamin D Status, VDR, and TLR Polymorphisms and Pulmonary Tuberculosis Epidemiology in Kazakhstan

**DOI:** 10.3390/nu16040558

**Published:** 2024-02-17

**Authors:** Dauren Yerezhepov, Aidana Gabdulkayum, Ainur Akhmetova, Ulan A. Kozhamkulov, Saule E. Rakhimova, Ulykbek Y. Kairov, Gulnur Zhunussova, Ruslan N. Kalendar, Ainur Akilzhanova

**Affiliations:** 1 Laboratory of Genomic and Personalized Medicine, Center for Life Sciences, National Laboratory Astana, Nazarbayev University, Astana 010000, Kazakhstan; 2 Laboratory of Bioinformatics and Systems Biology, Center for Life Sciences, National Laboratory Astana, Nazarbayev University, Astana 010000, Kazakhstan; 3 Institute of Genetics and Physiology, Almaty 020000, Kazakhstan; 4 Institute of Biotechnology HiLIFE, University of Helsinki, P.O. Box 65, 00014 Helsinki, Finland

**Keywords:** vitamin D deficiency, pulmonary tuberculosis, infectious disease, risk factors, epidemiology

## Abstract

Background: Tuberculosis (TB) and vitamin D deficiency remain major public health problems in Kazakhstan. Due to the high incidence of pulmonary tuberculosis in the country and based on the importance of vitamin D in the modulation of the immune response and the association of its deficiency with many health conditions, the aim of our research was to study the vitamin D status, VDR and TLR gene polymorphisms, and pulmonary tuberculosis epidemiology in Kazakhstan. Methods: A case-control study included 411 individuals diagnosed with pulmonary TB and 686 controls with no family history of pulmonary tuberculosis. Concentrations of serum vitamin D (25-(OH)D) levels were measured by electrochemiluminescence immunoassay. The gene polymorphisms were determined by real-time polymerase chain reaction (PCR) allelic discrimination assay using TaqMan probes. The association between the risk of pulmonary TB and polymorphisms was evaluated using multimodal logistic regression and assessed with the ORs, corresponding to 95% Cis, and the significance level was determined as *p* < 0.05. Results: 1097 individuals were recruited from 3 different regions of Kazakhstan. Biochemical data showed vitamin D deficiency (25-(OH)D < 20 ng/mL) was present in both groups, with the case group accounting for almost 95% and 43.7% in controls. Epidemiological data revealed that socioeconomic factors such as BMI < 25 kg/m^2^ (*p* < 0.001), employment (*p* < 0.001), diabetes (*p* < 0.001), and vitamin D deficiency (*p* < 0.001) were statistically different between case and control groups. Logistic regression analysis, adjusted by sex, age, BMI, residence, employment, smoking, alcohol consumption, and diabetes, showed that T/T polymorphism of the VDR gene (rs1544410, OR = 1.97, 95% CI: 1.04–3.72, *p* = 0.03) and A/A polymorphism of the TLR8 gene (rs3764880, OR = 2.44, 95% CI: 1.20–4.98, *p* = 0.01) were associated with a high risk of developing pulmonary tuberculosis. Conclusions: Vitamin D deficiency remains prevalent in our study cohort and is associated with TB progression. Socioeconomic determinants such as unemployment, BMI under 25 kg/m^2^, and diabetes are the main risk factors for the development of pulmonary TB in our study. A/A polymorphism of TLR8 (rs3764880) and T/T polymorphism (BsmI, rs1544410) of VDR genes may act as biomarkers for pulmonary tuberculosis in the Kazakh population.

## 1. Introduction

Tuberculosis (TB) is a complex and infectious disease caused by *Mycobacterium tuberculosis* (MTB) and is a major public health problem in many developing countries [1]. The World Health Organization (WHO) has identified the epidemiological and social risk factors that can influence the development of active forms of pulmonary tuberculosis. Such factors include contact with TB patients, smoking, maternity leave, HIV, drug abuse, alcohol consumption, and diabetes. In addition, the body mass index (BMI), which is beyond the normal ranges (18.5–24.99), is also a risk factor for many diseases, including tuberculosis. Unemployment is also a risk factor because the absence of income may lead to several health problems due to undernutrition or undernourishment [2,3]. TB incidence in low- and middle-income countries is high, where social and environmental factors are the main contributors [4]. Since migration has been increasing for the last 20 years and tends to take the direction from economically low to developed countries, it has become an essential factor in TB control [5].

It is estimated that over one-third of the world’s population is infected by MTB, but only 10% of the individuals can develop an active form of the disease. The remaining individuals will remain latently infected (LTBi) without clinical symptoms but can develop active disease in the future [6]. These data suggest that host genetics play an essential role in regulating the transition of LTBi to an active form, coupled with social and environmental factors. Bacille Calmette-Guérin (BCG) remains the only vaccine for TB. Unfortunately, this vaccine does not always protect from developing an active form of the disease [7].

Most humans get infected by inhaling MTB into the body’s airways. Inhaled MTB encounters one of the world’s most effective defensive systems—the immune system [8]. The first point that MTB encounters is alveolar macrophages. Engulfed MTB is recognized by pattern recognition receptors (PRR), which include toll-like receptors (TLRs), that trigger the synthesis of IL-1β and class A scavenger receptors (SR), SR-A/CD204, and MARCO, which bind with MTB by collagenous and SR cysteine-rich domains. Also, TLRs stimulated by lipopeptides from an infectious organism such as M. tuberculosis increase the expression of CYP27B1 and vitamin D receptor (VDR) genes in macrophages [9]. Escaped MTB induces the release of several substances, including pro-inflammatory cytokines such as interferon-γ (IFN-γ) that activate macrophages and intracellular mediators such as nitric oxide (NO) [10].

Vitamin D is a versatile molecule that modulates the immune system and plays an important role in the effectiveness of the immune response to various pathogens, including MTB. Many studies have found an association between vitamin D status and several health conditions, including cancers; bone development and remodeling; cardiovascular disorders (CVD); skin diseases (e.g., psoriasis); obesity, diabetes mellitus, and metabolic syndrome; and infections such as upper respiratory tract infection, influenza, HIV infection, bacterial vaginosis, and tuberculosis [11,12].

Vitamin D is a biologically inert molecule, and its activation requires two successive hydroxylation reactions. To enter the nucleus and perform its functions, the active form of vitamin D, 1,25-dihydroxyvitamin D, needs to bind to its receptor (VDR). One of these functions is the activation of m [13]. Various single nucleotide polymorphisms (SNPs) of the VDR gene have been studied, and some of them showed an association between susceptibility/resistance to tuberculosis [14,15,16]. TLRs play an important role in the immune response to MTB infection and the activation of the VDR gene. Consequently, variations in genes encoding these TLRs might alter the immune response [17]. There have been many studies on various SNPs of TLRs, and they showed varying results in different populations [18].

The population of Kazakhstan reached the mark of 20 million people on 14 November 2023. Kazakhstan is the ninth-largest country in the world by surface area and has one of the lowest population densities (7 per sq. km). Despite the low population density, the incidence of pulmonary TB (PTB) in Kazakhstan is the highest among CIS countries. In the last decade, TB incidence in Kazakhstan has declined from 227 per 100,000 individuals in 2014 to 81.7 per 100,000 individuals in 2018 and to 36.5 per 100,000 individuals in 2022, while mortality has increased from 8.4 to 15.2 per 100,000 individuals [19]. Kazakhstan has one of the highest numbers of multi-drug resistant TB (MDR-TB) patients in the world. Slightly more than a quarter of newly diagnosed TB patients and less than half of retreatment cases have MDR-TB [20].

The climate of Kazakhstan is mostly continental, with warm summers and cold winters. Despite having approximately 200 sunny days throughout the year on most of its territory, vitamin D deficiency is one of the major problems of Kazakhstan [21].

Due to the high incidence of pulmonary tuberculosis in the country and based on the importance of vitamin D in the modulation of the immune response and the association of its deficiency with many health conditions, the aim of our research was to study the vitamin D status, VDR and TLR gene polymorphisms, and pulmonary tuberculosis epidemiology in Kazakhstan.

## 2. Materials and Methods

*Study Subjects.* We conducted a case-control study. This study included 411 individuals diagnosed with active primary pulmonary TB. The group of controls consisted of 686 participants of Kazakh nationality with no family history of pulmonary tuberculosis. Recruitment took place in 3 regions of Kazakhstan. Diagnosis of the case group was confirmed by recent radiographic evidence of TB cavitation and positive laboratory data (smear/culture/TB-DNA). The case group participants were recruited at local TB dispensaries. The control subjects were matched by sex, age, ethnicity, and residence. Individuals with immunodeficiency-associated diseases, hepatitis virus infection, and other lung and chronic respiratory diseases were excluded from this study. Persons who had supplements containing vitamin D or its combinations (including multivitamins) one year prior to recruitment were excluded from the study to avoid intervention in serum vitamin D levels. Since seasonal serum vitamin D levels can vary due to different exposures to sunlight, recruitment was performed during the warm season for all three regions between May and September 2021. In 2022, we did the recruitment process in the same period to avoid differences in blood biochemical parameters. Additionally, case and control groups were recruited in the same geographical area.

All subjects were unrelated Kazakhs, older than 18 years, with available clinical data. Demographic data and clinical information of the participants were reviewed from the medical records.

Eligible participants filled out structured sociodemographic and clinical questionnaires during an in-person interview, and anthropometric measurements, including measurements of height, weight, and BMI, were measured at the same time. Medical data were taken from the medical records database of local hospitals.

The study was conducted in accordance with the Declaration of Helsinki and approved by the Local Ethics Committee of the Private Institution “National Laboratory Astana” (Protocol N04-2020, 26 August 2020). All subjects signed a written informed consent form. 

*Biochemical analysis.* Concentrations of serum 25-(OH)D of the case group were performed at local TB dispensaries after at least 12 h of night fasting. Biochemical analysis of patients was performed at centralized biochemical laboratories in the respective regions. Concentrations of serum 25-(OH)D levels of all participants representing the group of cases were measured by electrochemiluminescence immunoassay using a Roche Cobas vitamin D total assay reagent kit according to the manufacturer’s protocol. Blood sampling of case group participants was performed between 8:00 and 10:00 a.m. after 12 h of fasting. Blood sampling of the control group was performed under the same conditions as that of the case group. Blood samples of the control group were drawn into test tubes for subsequent centrifugation and storage at −80 °C until they were used for analysis. Since the results of different methods and kits used to measure the serum 25-(OH)D levels can vary, the serum 25-(OH)D levels of the control group were measured by the same kit as for the cases in order to avoid potential biases in serum 25-(OH)D levels. The Roche Cobas vitamin D total assay reagent kit is an electrochemiluminescence protein-binding immunoassay for quantitative determination of total 25-(OH)D in human serum and plasma. The 27-min-long assay consists of three steps of incubation: Pretreatment, incubation with ruthenium-labeled VDBP, and addition of 25-(OH)D labeled with biotin and streptavidin-coated microparticles. Vitamin D deficiency has been defined as a 25-(OH)D of less than 20 ng/mL, according to international recommendations [22].

*DNA Isolation.* Blood was collected into tubes containing K2EDTA. DNA was extracted using the QIAamp DNA Mini extraction Kit (Qiagen GmbH, Hilden, Germany), according to the manufacturer’s instructions for DNA purification from whole blood, and stored at −20 °C. The quantity and quality of the DNA were evaluated using the NanoDrop 2000 UV spectrophotometer. The exact quantity was measured by the Qubit BR Assay Kit (ThermoFisher Scientific, Waltham, MA, USA) on a Qubit v2.0 fluorometer. DNA integrity was tested on 1% agarose gel electrophoresis. DNA was stored at −20 °C in the Laboratory Biobank. 

*Genotyping.* TLRs play a major role in the activation of VDR gene expression in macrophages, as they take part in the primary immune response during the first encounter with *M. tuberculosis*. Alterations in the amino acid sequence of TLR proteins can affect the expression pattern of the VDR gene. SNPs of the VDR gene itself can affect the binding pattern with its substrate. In order to investigate the role of TLR and VDR polymorphisms in susceptibility to PTB, we chose the polymorphisms [13,14,15,16,17,18], which showed statistical significance in many populations. Polymorphisms of selected genes are presented in Table 1.

The gene polymorphisms were determined by real-time polymerase chain reaction (PCR) allelic discrimination assay using TaqMan probes (Applied Biosystems, Foster City, CA, USA) on 7900 HT Fast Real-Time PCR System following the manufacturer’s protocol. The 10 μL reaction mixture consisted of 5 uL of 2× TaqMan™ Genotyping master mix (Applied Biosystems, Foster City, CA, USA), 0.25 uL of 40× (catalog number: 4351379, Applied Biosystems, Foster City, CA, USA), or 0.5 uL of 20× (catalog number: 4331349, Applied Biosystems, Foster City, CA, USA) TaqMan™ Probe, and 10 ng of genomic DNA.

The genotype distribution was analyzed using SDS v2.4 software (Applied Biosystems, Foster City, CA, USA). Each genotyping was repeated three times to avoid mistakes during software distribution. Minor allele frequencies less than 0.05 were excluded from statistical analysis.

*Statistical Analysis.* Quantitative variables were expressed as a result (±standard deviation) with a normal distribution. We applied the Hardy–Weinberg equilibrium test for all subjects, cases, and control groups separately. A 2 × 2 association between the risk of pulmonary TB, risk factors, and vitamin D levels was analyzed using the Pearson χ^2^ test. The association between the risk of pulmonary TB and polymorphisms was evaluated using multimodal (genotypic, dominant, recessive, and overdominant) logistic regression and assessed with the ORs and their corresponding 95% CIs. We defined the models as follows: Genotypic (AA vs. Aa vs. aa), dominant (AA + Aa vs. aa), recessive (AA vs. Aa + aa), and overdominant (AA + aa vs. Aa), where the major and the minor alleles are A and a, respectively. All the tests were 2-sided, with a significance level of *p* < 0.05, and were estimated using SPSS 25 (IBM, Armonk, NY, USA) software.

## 3. Results

**Figure 1:**
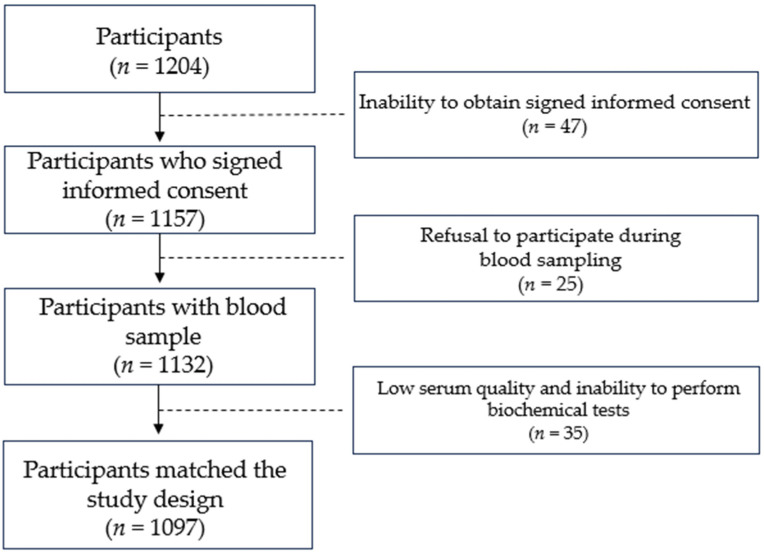
Illustration of study design.

*Study group*. Twelve hundred and four unrelated individuals of Kazakh nationality were recruited for the study. Forty-seven individuals were excluded due to their inability to obtain signed informed consent. The following individuals of the case group refused to participate during blood sampling: Two HIV-positive persons, one drug abuser, an 84-year-old patient, and nine women on maternity leave. Twelve individuals from the control group also refused to participate during blood sampling. Yet another 35 individuals from both groups were removed due to low serum quality and an inability to perform biochemical tests. The final study group consisted of 411 individuals diagnosed with active primary PTB and 686 participants of Kazakh nationality with no family history of TB. The response rate in the case and control groups was 58.2% and 97.9%, respectively. The study design is shown in Figure 1.

### 3.1. Epidemiology of Pulmonary TB in Kazakhstan

*Demographic characteristics* and *epidemiological data.* At enrollment, the median age was 38.4 ± 13.8 years (18–76), median height was 166.7 ± 8.5 cm, weight was 64.0 ± 12.7 kg, and BMI was 22.9 ± 4.1. Females were slightly over half of the participants (*n* = 570, 52% female; *n* = 527, 48% male). The study group consisted of 411 cases of primary pulmonary TB and 686 controls with no family history of TB (Table 2). All participants had BCG scars. Recruitment took place in three different climate zones with different population densities in Kazakhstan [20]. As seen in Table 2, participants were recruited according to incidence rate and population density.

Slightly more than 40% of the study group were living in urban locations, 586 (53.4%) of participants were unemployed, alcohol consumption was a little over 1%, close to 30% were obese (BMI > 25) or overweight (BMI > 30), 35 participants (3.2%) had diabetes with prevalence in the case group, 28 cases contacted with TB patients, with none in the control group, and only 9% of study participants were active smokers or had smoked previously for more than 10 years.

The overall mean serum 25-(OH)D level of the study group was 20.4 ± 9.7 ng/mL, with 12.9 ± 3.8 and 24.8 ± 3.1 ng/mL in the case and control groups, respectively. The mean level of serum 25-(OH)D in the control group was twice that of the case group. North region individuals had the lowest serum 25-(OH)D levels (16.0 ± 9.2 ng/mL), and South-East had the highest (22.4 ± 10.1 ng/mL). Almost 63% of all participants had vitamin D deficiency (≤20.00 ng/mL), and 94.9% of the case group were in deficiency ranges, while the control group had 43.7%.

*Epidemiological Risk Factors of Pulmonary TB.* Several risk factors for tuberculosis incidence were identified (Table 3). Male individuals had a higher incidence rate of tuberculosis (54.5% among males versus 45.5% among females). More cases were living in rural regions of Kazakhstan (242 vs. 169), 1.5% and 0.9% of cases and control groups drank alcohol on a regular basis, respectively. The distribution of smokers among both groups was almost identical (9.7% and 8.6%). None of the control groups were contacted with TB. Only 14.4% of the case group were overweight or obese (vs. 39.2% in controls). However, indicators such as unemployment and diabetes showed dramatic differences in the case and control groups (70.6% vs. 43.1% and 7.5% vs. 0.5%, respectively).

Several risk factors such as alcohol consumption, smoking, and type of residence showed no association with pulmonary tuberculosis in our study group. However, BMI (*p* < 0.001), employment status (*p* < 0.001), diabetes (*p* < 0.001), and serum 25-(OH)D levels (*p* < 0.001) contribute a lot to developing pulmonary tuberculosis. This means unemployed individuals with a low BMI and diabetes have a very high chance of developing pulmonary tuberculosis.

### 3.2. Genetics of Pulmonary Tuberculosis in Kazakhstan

*Genes and polymorphisms.* All samples were successfully sequenced for SNPs. All SNPs were verified by triple repeat, and no deviations were detected. All SNPs, but TLR2, had more than 5% of minor alleles. Genotyping results are shown in Table 4. The observed genotype frequencies did not deviate from the expected frequency values according to the Hardy–Weinberg equilibrium test. The minor allele of the TLR2 gene was absent in our cohort, and we didn’t take it to statistical analysis.

*Association between Pulmonary Tuberculosis and Polymorphisms in the Studied Genes.* The association between pulmonary tuberculosis and studied polymorphisms was evaluated through various analytical models: genotypic or codominant, dominant, recessive, and overdominant with no adjustment and adjusted by sex, age, BMI, residence, employment, smoking, alcohol consumption, and diabetes (Table 5).

All four polymorphisms of the VDR gene did not show a statistically significant association. Their association did not change after adjustment. Crude analysis revealed a statistically significant association between A/A polymorphisms of the TLR8 gene (OR = 2.07, 95% CI = 1.09–3.94, *p* = 0.01). Heterozygous A/G polymorphisms of the TLR8 gene (OR = 1.33, 95% CI = 1.01–1.75, *p* = 0.01) also showed a statistically significant association. After adjustment by sex, age, BMI, residence, employment, smoking, alcohol consumption, and diabetes, an association of both A/A and A/G polymorphisms of the TLR8 gene (OR = 2.56, 95% CI = 1.25–5.24, *p* = 0.02 and OR = 1.18, 95% CI = 0.84–1.65, *p* = 0.02, respectively) remained significant after adjustment.

The association between pulmonary tuberculosis and studied polymorphisms evaluated in dominant, recessive, and overdominant models with crude analysis and adjusted by sex, age, BMI, residence, employment, smoking, alcohol consumption, and diabetes is shown in Table 6. In the unadjusted dominant model, TLR8 rs3764880 A allele was associated with a higher risk of developing pulmonary TB (OR = 1.41, 95% CI: 1.09–1.83, *p* = 0.001, GG vs. G/A-A/A). In the unadjusted recessive model, carriers of the TLR8 rs3764880 A/A (OR = 1.90, 95% CI: 1.00–3.59, *p* = 0.04, G/G-A/G vs. A/A) genotype were associated with a higher risk of developing pulmonary TB. In the unadjusted overdominant model, the SNPs studied did not show a statistically significant association.

The evaluation of the association between pulmonary tuberculosis and studied polymorphisms in dominant, recessive, and overdominant models with adjustment by sex, age, BMI, residence, employment, smoking, alcohol consumption, and diabetes showed no statistically significant association in dominant and overdominant models. The association of the TLR8 gene (rs3764880) in the recessive model (OR = 2.44, 95% CI: 1.20–4.98, *p* = 0.001) remained statistically significant. Interestingly, BsmI (rs1544410) polymorphism of the VDR gene showed no significant association in the codominant model (OR = 0.82, 95% CI: 0.61–1.11, *p* = 0.2). However, in the recessive model, the association entered 0.05 *p*-value limits (OR = 1.97, 95% CI: 1.04–3.72, *p* = 0.03).

## 4. Discussion

Tuberculosis remains one of the leading causes of death by a single infectious agent [1]. If high-income countries account for not more than 10 cases per 100,000 population per year, low-income and middle-income countries have an incidence of little less than 200 cases per 100,000 population per year, with several low-income countries accounting for up to 400 cases per 100,000 annually [23].

Kazakhstan is a large country located between two continents and has a continental climate in most parts. Kazakhs represent the majority (70.4%) of the population of Kazakhstan. Russians, Uzbeks, Ukrainians, and Uighurs account for 15.6%, 3.2%, 2.0%, and 1.5%, respectively. Other nationalities fill in the remaining 7.3% [24]. We recruited participants of Kazakh nationality because Kazakhs represent the majority of the population of Kazakhstan and to minimize the heterogeneity in BMI, body composition, metabolism, and genetics since Asians and Caucasians differ by the indicated parameters [25,26]. Also, we tried to minimize selection bias by recruiting only individuals of Kazakh nationality. However, a response rate of 58.2% in the case group vs. 97.9% in the controls in our study still might increase selection bias since not all participants accepted the invitation for recruitment [27]. Participation rates for case-control studies have declined over the past several decades, and this rate depends on several factors. Individuals with a higher socioeconomic status, a higher education, and current employment are more likely to agree to participate in case-control or epidemiologic studies. Additionally, response rates are higher in women than in men. A lower response rate is typical for the case groups, especially when the case is a health condition with a heavy emotional background [28]. Our study’s control group is almost twice as large as the case group. Many studies have included unequal numbers of cases with controls, such as 2:1 or 4:1, to increase the power of the study [29]. Females are slightly overrepresented in controls (2 control individuals vs. 1 case participant), while males are underrepresented (1.5 vs. 1), which can be the subject of potential biases. Also, we had the lowest recruitment rate in Northern Kazakhstan since Kazakhs represent only 44% of the population (the majority is Russians—47%), while the southeast and southwest parts of Kazakhstan account for over 70% of Kazakhs [30].

Only recently was Kazakhstan given a middle-income country status. Still, risk factors for TB remain unchanged: Contact and duration with a person who has an infectious form of TB, health conditions such as low BMI, diabetes, HIV, immune-system-compromising diseases, smoking, drug abuse, pregnancy and maternity leave, and unemployment, which might lead to undernutrition [31,32,33,34]. In our study, socioeconomic determinants such as unemployment and health conditions such as diabetes and low BMI are the main social risk factors for PTB, confirming previous studies in different countries [31,34]. It is important to mention that almost 59% of the cases were in rural regions. The higher incidence rate is probably due to the low number of hospitals and medical organizations in rural regions. In addition, because of the large area of Kazakhstan, some remote settlements are very far from medical organizations. Also, the income rate in rural regions is usually lower than in urban areas. Due to these circumstances, the population of rural regions might self-medicate more often than urban residents when observing the primary symptoms of TB, which are similar to flu or other respiratory infections. This might be the reason for the higher incidence rate. Recent data indicate that many health conditions, including TB, are affecting adolescents and young adults [35]. In our case group, we recruited individuals who were 18 years and older. Slightly less than one-tenth of the case group (41) were students aged between 18 and 29 years old who were diagnosed with primary pulmonary TB and studied in three different regions of Kazakhstan. Undergraduate students mostly enter university at 16–17, so we might assume that they are infected in university surroundings because entering university requires a medical examination, including a chest X-ray.

Vitamin D deficiency has been considered a global health issue [36]. Many studies have reported that vitamin D deficiency is associated with bone hypomineralization, diabetes, hypertension, metabolic syndrome, cancers, cardiovascular diseases, upper respiratory tract infections, and infectious diseases, including TB [37,38,39,40,41,42,43,44].

Our study revealed a high level of vitamin D deficiency in a studied group. Vitamin D deficiency is highest in northern Kazakhstan due to colder climate conditions, with five to six months of cold and snow and shorter sunlight periods. However, this region accounts for 200 sunny days per year (the average latitude is 53°12′51.66″). However, during the relatively long frost season, people must wear cold-season clothes longer, exposing only their faces to the sunshine. The southeast part of Kazakhstan is densest by population and is a high-humid mountain region, subsequently having a higher UV index and a 7-month warm (up to +40 °C in summer) period. Subsequently, vitamin D levels in individuals who are recruited in southeast Kazakhstan are higher than in north Kazakhstan. However, relatively few participants representing the southeast part of Kazakhstan have sufficient vitamin D (>30 ng/mL) levels in serum, even in the control group. Almaty (capital 1929–1999) is the largest city and the financial center of Kazakhstan. One of the reasons for vitamin D deficiency might be that people spend more hours in offices. The southwest part is the hottest part of Kazakhstan, with up to 4 months of warm and four months of hot weather and a high population density. Despite that, the southwest region is more agriculture-oriented, and people should spend more time in the sunshine. Serum 25-(OH)D levels should be higher. Still, serum 25(OH)D levels remain insufficient, even in most of the participants of our control group.

Not much research has been carried out regarding vitamin D levels in the population of Kazakhstan. To date, Gromova et al. have investigated vitamin D deficiency (VDD) in the Kazakhstani population and reported a high level of VDD [21]. Several research works investigated associations between vitamin D status and human immunodeficiency virus (HIV) [45], skin disorders [46], arterial hypertension [47], depression [48], and COVID-19 susceptibility [49]. All these research studies showed a median serum 25-(OH)D level over 20 ng/mL. Zhumina et al. investigated vitamin D levels in leukemia patients in Central Kazakhstan. They reported low vitamin D levels in leukemia patients, and the median of vitamin D in the control group was slightly over the deficiency limit (21.1 ng/mL) [50]. Natural dietary sources of vitamin D are oily fish and cod liver oil. However, the traditional diet of Kazakhs includes meat and poultry, vegetables, flour, and dairy products. The western part of Kazakhstan is laid on the shores of the Caspian Sea, the largest lake in the world, with oceanic salty water and ocean animals and fish species. The population of west Kazakhstan has more access to natural sources of vitamin D. Also, Aktau is the only relatively large city located directly on the shore of the Caspian Sea with exceptional climatic conditions.

Interpretation of any findings about vitamin D levels needs to be done accurately since vitamin D levels depend on multiple factors and are prone to potential biases. The vitamin D molecule is stable when bound to vitamin D-binding proteins and circulates in the body for 2–3 weeks [50]. Vitamin D levels depend on sex, age, diet, season, place of residence, duration of UV exposure and strength of the UV rays, physical activity, genetics, and even the amount of pigment in the skin [51]. The popularity of daily cosmetics that protect from UV overexposure (sun protection factor—SPF) also reduces sufficient exposure to sunlight.

Other limitations are the sample state and assay used to measure vitamin D levels. All the biochemical measurements are preferably done from fresh serum or plasma. However, many research studies freeze serum samples and store them until further use, so serum undergoes at least a single freeze–thaw cycle. Wielder et al. reported the stability of vitamin D molecules even in unprocessed blood samples. The authors tested several conditions, such as storage for several days at room temperature and in a refrigerator, several freeze-thaw cycles, and storage at −20 °C for up to 2 months. They reported a 2.6% increase in vitamin D concentration after a single freeze-thaw cycle and a decrease in concentration of 4% during storage at −20 °C for up to 2 months [52]. However, Abraham et al. reported a 12% reduction in vitamin D concentrations following the single freeze-thaw cycle [53]. The assays used to measure vitamin D levels also pose some potential controversies. Tandem mass spectrometry has been recognized as the gold standard due to its higher precision and separate detection of the D_2_ and D_3_ isoforms of vitamin D. However, its cost blocks its widespread utilization. Currently, immunoassays are commonly used to test the serum’s vitamin D concentrations. Abdel-Wareth et al. compared the vitamin D total electrochemiluminescence protein binding assay (Roche Diagnostics, Manheim, Germany) with the HPLC method and reported an 8–10.4% bias (negative) in concentrations of both D_2_ and D_3_ isoforms of vitamin D [54].

Even considering all the potential factors influencing vitamin D levels and given that the blood samples were collected during warm periods, our group will have a greater score of VDD in cold periods. Our results suggest that most of the population we studied requires vitamin D supplementation. However, this must be performed accordingly, only with medical advice and in an individualized manner.

Currently, about 30% of the world’s population is infected with *M. tuberculosis*. The risk of becoming active during a lifetime is approximately 5–10%. These numbers indicate the genetic component of human susceptibility to active and latent tuberculosis [18]. It has been reported that host–pathogen interactions play an important role in disease progression [55].

Genes involved in the innate immune response represent an interdependent cascade of interactions when an alteration in one chain link can disrupt its integrity. The main function of this cascade of reactions is to trap the invaded infectious agent and eliminate it as soon as possible. The elimination of bacteria at first encounter is very important. So, genes involved in the primary innate immune response have been targeted for much research. Alveolar macrophages are responsible for the primary recognition of *M. tuberculosis* by PRRs, which activate the engulfment process and signals to induce the expression of genes of many antimicrobial agents and intracellular mediators, such as cytokines and nitric oxide, including VDR [9].

TLRs play an important role in the immune response to the MTB infection and the activation of the VDR gene. Any alterations in the amino acid sequence of TLRs might decrease the immune response since they can affect the recognition pattern of receptors and decrease their affinity. There have been many studies on various SNPs of TLRs, and they showed varying results in different populations [18]. Currently, a single study is dedicated to TLRs in the population of Kazakhstan [56].

Vitamin D receptor (VDR) is known as an innate immune response mediating transcription factor. VDR enhances the expression of several antimicrobial peptides and, when bound to vitamin D, can induce the secretion of anti-inflammatory cytokines and promote autophagy [18]. The VDR gene has multiple SNPs. Much of the research work focused on the four main SNPs—FokI, TaqI, ApaI, and BsmI. The association of these SNPs was tested for various health conditions in many populations, including TB [57,58,59,60]. To date, a single research report has been published on the association of VDR polymorphisms with susceptibility to TB in the Kazakh population. However, their findings did not show a statistically significant association (*p*-values for all 4 SNPs > 0.05).

In this study, we tried to find the association of polymorphisms of TLR2, TLR8, and four polymorphisms of the VDR gene (FokI, TaqI, ApaI, and BsmI) with pulmonary TB progression in the Kazakh population. In the case of TLR2 polymorphism (rs1898830), we could not detect any minor allele, so we could not perform association analysis. The TLR2 gene has a very low minor allele frequency (MAF < 0.01), and several of its polymorphisms are absent in different populations [61]. A/A and A/G polymorphisms of the TLR8 gene showed a statistically significant association for TB susceptibility in co-dominant and recessive models. Our findings confirm the results of the previous studies performed on a larger combined population [62]. Unfortunately, we did not find a statistically significant association between any of the four VDR polymorphisms and TB in the co-dominant model, where the contribution of each of the genotypes was assessed equally. However, BsmI (rs1544410) polymorphism of the VDR gene showed a statistically significant association in the recessive model (OR = 1.97, 95% CI: 1.04–3.72, *p* = 0.03), meaning T/T homozygous genotypes increase susceptibility to TB almost twice in our study group.

The phenotypic diversity of the Kazakh population might indicate genetic admixture [63,64]. In genetic association studies in admixed populations, genomic ancestry should be investigated to avoid misinterpretation due to the population substructure [65]. Any genetic research must consider the potential biases that might arise, but the genotyping of our study group did not show any deviations in the distribution of genotypes of selected genes from previous studies [14,15,16,17,18]. There is a lack of genomic data on the ancestry of the Kazakh population, but tracking of ancestry by paternity line is performed by the so-called “Shezhire”, and most of the people know the names of their previous seven grandfathers and the tribe they belong to [66]. All recruited individuals knew at least their three grandfathers by paternal and maternal lines. This minimized the genetic admixture and heterogeneity of genetic data.

However, these results must be interpreted with caution, given the need for more scientific evidence to enable us to determine which of these SNPs could be used as biomarkers for the risk of developing pulmonary tuberculosis. Continued research with a larger cohort is still needed. Sample homogeneity and a relatively large cohort are the strengths of our research because only representatives of Kazakh nationality were recruited for the study.

### Limitations

There were several limitations in the present study. First, there is uneven representation of both case and control groups in all three regions, with some underrepresented. The low response rate in the case group (58.2%) could be subjected to potential selection biases. Second, serum 25-(OH)D levels depend on multiple factors, and potential biases must be taken into consideration. Third, in the present study, we could not obtain data for the latent TB status of all participants and did not evaluate the genetics of the probability of progression of the latent TB to an active form. In future studies, all these factors should be improved or strengthened.

## 5. Conclusions

Vitamin D deficiency remains prevalent in our study cohort and is associated with TB progression. Socioeconomic determinants such as unemployment, BMI under 25 kg/m^2^, and diabetes are the main risk factors for the development of pulmonary TB in our study. A/A polymorphism of TLR8 (rs3764880) and T/T polymorphism (BsmI, rs1544410) of VDR genes may act as biomarkers for pulmonary tuberculosis in the Kazakh population.

## Figures and Tables

**Table 1:** Polymorphisms of selected genes.

Gene	Name	Reference Number	Context Sequence
VDR	FokI	rs2228570	GGAAGTGCTGGCCGCCATTGCCTCC**[A/G]** TCCCTGTAAGAACAGCAAGCAGGCC
	TaqI	rs731236	TGGACAGGCGGTCCTGGATGGCCTC**[A/G]** ATCAGCGCGGCGTCCTGCACCCCAG
	BsmI	rs1544410	GAGCAGAGCCTGAGTATTGGGAATG**[T/C]** GCAGGCCTGTCTGTGGCCCCAGGAA
	ApaI	rs7975232	AAGGCACAGGAGCTCTCAGCTGGGC**[A/C]** CCTCACTGCTCAATCCCACCACCCC
TLR2	A-G	rs1898830	ATAGTAAAATAAATCCAGAGAAATC**[A/G]** GAACAGGGGAAATAATAATATAAGA
TLR8	A-G	rs3764880	AATGAAAAATTAGAACAACAGAAAC**[A/G]** TGGTAAGCCACTTCTATTTCTTTAG

TLR2: toll-like receptor 2; TLR8: toll-like receptor 8; VDR: vitamin D receptor.

**Table 2:** Demographic and epidemiological data of study participants (*n* = 1097).

Details	All (*n* = 1097)	Case (*n* = 411)	Controls (*n* = 686)
Age, mean ± SD, years	38.4 ± 13.8	35.0 ± 13.1	40.5 ± 13.9
Height, mean ± SD, cm	166.7 ± 8.5	166.6 ± 8.9	166.7 ± 8.3
Weight, mean ± SD, kg	64.0 ± 12.7	58.6 ± 10.9	67.2 ± 12.5
BMI, mean ± SD, kg/m^2^	22.9 ± 4.1	21.0 ± 3.6	24.1 ± 4.0
Gender, *n* (%)			
Male	527 (48)	224 (54.5)	303 (44.2)
Female	570 (52)	187 (45.5)	383 (55.8)
Residence region, *n* (%)			
North	200 (18.2)	80 (19.5)	120 (60)
South-East	457 (41.7)	172 (41.8)	285 (62.4)
South-West	440 (40.1)	159 (38.7)	281 (63.9)
Risk factors, *n* (%)			
Residence			
Urban	449 (40.9)	169 (41.1)	280 (40.8)
Rural	648 (59.1)	242 (58.9)	406 (59.2)
Employment			
Yes	511 (46.6)	121 (29.4)	390 (56.9)
No	586 (53.4)	290 (70.6)	296 (43.1)
Alcohol consumption			
Yes	12 (1.1)	6 (1.5)	6 (0.9)
No	1085 (98.9)	405 (98.5)	608 (99.1)
BMI, kg/m^2^			
<24.99	769 (70.1)	352 (85.6)	417 (60.8)
>25.00	328 (29.9)	59 (14.4)	269 (39.2)
Diabetes			
Yes	35 (3.2)	31 (7.5)	4 (0.6)
No	1062 (96.8)	380 (92.5)	682 (99.4)
Contact with TB			
Yes	28 (2.5)	28 (6.8)	0
No	1069 (97.5)	383 (93.2)	686 (100)
Smoking			
Yes	99 (9.0)	40 (9.7)	59 (8.6)
No	998 (91.0)	371 (92.3)	682 (91.4)
Serum 25-(OH)D, mean ± SD, ng/mL			
Serum 25-(OH)D overall	20.4 ± 9.7	12.9 ± 3.8	24.8 ± 3.1
Serum 25-(OH)D by regions			
North	16.0 ± 9.2	9.6 ± 3.4	20.3 ± 3.8
South-East	22.4 ± 10.1	14.3 ± 5.6	26.6 ± 3.1
South-West	20.3 ± 9.5	13.0 ± 2.1	23.4 ± 2.8
Serum 25-(OH)D, ng/mL			
>20.01	407 (37.1)	21 (5.1)	360 (56.3)
≤20.00	690 (62.9)	390 (94.9)	326 (43.7)

BMI: body mass index; SD: standard deviation; TB: tuberculosis.

**Table 3:** Risk factors for developing pulmonary tuberculosis (*n* = 1097).

Risk Factors		Case	Control	*p*-Value *
Alcohol consumption	Yes	6	6	0.6
	No	405	680	
Diabetes	Yes	31	4	<0.001
	No	380	682	
Smoking	Yes	40	59	0.6
	No	371	682	
BMI, kg/m^2^	<24.99	352	417	<0.001
	>25.00	59	269	
Contact with TB	Yes	28	0	<0.001
	No	383	686	
Residence	Urban	169	280	1.0
	Rural	242	406	
Employment	Yes	121	390	<0.001
	No	290	296	
Serum 25-(OH)D	>20 ng/mL	21	417	<0.001
	<20 ng/mL	390	269	

* *p*-values derived by χ^2^ test for comparison of the case and control groups. BMI: body mass index; TB: tuberculosis.

**Table 4:** Data on polymorphisms of selected genes.

Gene	Polymorphism	Reference Number	Genotype	Amount	%
VDR	FokI	rs2228570	G/G	477	0.43
			A/G	488	0.44
			A/A	132	0.12
VDR	TaqI	rs731236	A/A	631	0.58
			A/G	396	0.36
			G/G	70	0.06
VDR	BsmI	rs1544410	C/C	627	0.57
			C/T	410	0.37
			T/T	60	0.05
VDR	ApaI	rs7975232	A/A	221	0.2
			C/A	532	0.48
			C/C	344	0.31
TLR2	G-A	rs1898830	G/G	1064	0.97
			G/A	33	0.03
			-	-	-
TLR8	G-A	rs3764880	A/A	175	0.16
			G/A	379	0.35
			G/G	543	0.49

TLR2: toll-like receptor 2; TLR8: toll-like receptor 8; VDR: vitamin D receptor.

**Table 5:** Association between pulmonary tuberculosis and studied polymorphisms.

Polymorphisms	Geno-Type	Case	Control	C-OR (95% CI)	*p*-Value	A-OR * (95% CI)	*p*-Value
VDR FokI	G/G	184 (44.8%)	293 (42.7%)	1.00	0.6	1.00	0.3
	A/G	175 (42.6%)	313 (45.6%)	1.12 (0.87–1.46)		1.17 (0.86–1.59)	
	A/A	52 (12.7%)	80 (11.7%)	0.97 (0.65–1.43)		0.82 (0.52–1.30)	
VDR TaqI	A/A	236 (57.4%)	395 (57.6%)	1.00	0.7	1.00	0.7
	A/G	152 (37%)	244 (35.6%)	0.96 (0.74–1.24)		0.97 (0.71–1.31)	
	G/G	23 (5.6%)	47 (6.8%)	1.22 (0.72–2.06)		1.19 (0.64–2.19)	
VDR BsmI	C/C	236 (57.4%)	391 (57%)	1.00	0.5	1.00	0.1
	C/T	157 (38.2%)	253 (36.9%)	0.97 (0.75–1.26)		1.04 (0.77–1.40)	
	T/T	18 (4.4%)	42 (6.1%)	1.41 (0.79–2.50)		1.99 (1.04–3.81)	
VDR ApaI	C/C	116 (28.2%)	228 (33.2%)	1.00	0.2	1.00	0.4
	A/C	204 (49.6%)	328 (47.8%)	0.82 (0.62–1.09)		0.83 (0.60–1.16)	
	A/A	91 (22.1%)	130 (18.9%)	0.73 (0.51–1.03)		0.76 (0.51–1.15)	
TLR8	G/G	288 (70.1%)	428 (62.4%)	1.00	0.01	1.00	0.02
	A/G	110 (26.8%)	218 (31.8%)	1.33 (1.01–1.75)		1.18 (0.84–1.65)	
	A/A	13 (3.2%)	40 (5.8%)	2.07 (1.09–3.94)		2.56 (1.25–5.24)	

* Adjusted by sex, age, BMI, residence, employment, smoking, alcohol consumption, and diabetes; A-OR: adjusted odds ratio; CI: confidence interval; C-OR: crude odds ratio; TLR8: toll-like receptor 8; VDR: vitamin D receptor.

**Table 6:** Different models of association between pulmonary tuberculosis and polymorphisms in the studied genes.

Polymorphisms	Model	Genotype	Case	Control	C-OR (95% CI)	*p*-Value	A-OR * (95% CI)	*p*-Value
VDR FokI	Dominant	G/G	184 (44.8%)	293 (42.7%)	1.00	0.5	1.00	0.6
		A/G-A/A	227 (55.2%)	393 (57.3%)	1.09 (0.85–1.39)		1.08 (0.81–1.45)	
	Recessive	G/G-A/G	359 (87.3%)	606 (88.3%)	1.00	0.6	1.00	0.2
		A/A	52 (12.7%)	80 (11.7%)	0.91 (0.63–1.32)		0.76 (0.49–1.16)	
	Overdominant	G/G-A/A	236 (57.4%)	373 (54.4%)	1.00	0.3	1.00	0.2
		A/G	175 (42.6%)	313 (45.6%)	1.13 (0.88–1.45)		1.23 (0.92–1.64)	
VDR TaqI	Dominant	A/A	236 (57.4%)	395 (57.6%)	1.00	1.0	1.00	1.0
		A/G-G/G	175 (42.6%)	291 (42.4%)	0.99 (0.78–1.27)		1.00 (0.74–1.33)	
	Recessive	A/A-A/G	388 (94.4%)	639 (93.2%)	1.00	0.4	1.00	0.6
		G/G	23 (5.6%)	47 (6.8%)	1.24 (0.74–2.08)		1.20 (0.66–2.20)	
	Overdominant	A/A-G/G	259 (63%)	442 (64.4%)	1.00	0.6	1.00	0.7
		A/G	152 (37%)	244 (35.6%)	0.94 (0.73–1.21)		0.95 (0.71–1.28)	
VDR BsmI	Dominant	C/C	236 (57.4%)	391 (57%)	1.00	0.1	1.00	0.4
		C/T-T/T	175 (42.6%)	295 (43%)	0.79 (0.60–1.03)		1.13 (0.84–1.51)	
	Recessive	C/C-C/T	393 (95.6%)	644 (93.9%)	1.00	0.2	1.00	0.03
		T/T	18 (4.4%)	42 (6.1%)	0.82 (0.61–1.11)		1.97 (1.04–3.72)	
	Overdominant	C/C-T/T	254 (61.8%)	433 (63.1%)	1.00	0.6	1.00	0.9
		C/T	157 (38.2%)	253 (36.9%)	0.93 (0.73–1.19)		0.99 (0.73–1.33)	
VDR ApaI	Dominant	C/C	116 (28.2%)	228 (33.2%)	1.00	0.9	1.00	0.2
		A/C-A/A	295 (71.8%)	458 (66.8%)	1.02 (0.79–1.30)		0.81 (0.60–1.10)	
	Recessive	C/C-A/C	320 (77.9%)	556 (81%)	1.00	0.2	1.00	0.38
		A/A	91 (22.1%)	130 (18.9%)	1.42 (0.81–2.51)		0.85 (0.60–1.21)	
	Overdominant	C/C-A/A	207 (50.4%)	358 (52.2%)	1.00	0.7	1.00	0.6
		A/C	204 (49.6%)	328 (47.8%)	0.95 (0.73–1.22)		0.93 (0.70–1.23)	
TLR8	Dominant	G/G	288 (70.1%)	428 (62.4%)	1.00	0.001	1.00	0.1
		A/G-A/A	123 (29.9%)	258 (37.6%)	1.41 (1.09–1.83)		1.34 (0.97–1.84)	
	Recessive	G/G-A/G	398 (96.8%)	646 (94.2%)	1.00	0.04	1.00	0.001
		A/A	13 (3.2%)	40 (5.8%)	1.90 (1.00–3.59)		2.44 (1.20–4.98)	
	Overdominant	G/G-A/A	301 (73.2%)	468 (68.2%)	1.00	0.8	1.00	0.6
		A/G	110 (26.8%)	218 (31.8%)	1.27 (0.97–1.67)		1.10 (0.79–1.54)	

* Adjusted by sex, age, BMI, residence, employment, smoking, alcohol consumption, and diabetes; A-OR: adjusted odds ratio; CI: confidence interval; C-OR: crude odds ratio; TLR8: toll-like receptor 8; VDR: vitamin D receptor.

## Data Availability

The data presented in this study are available on request from the corresponding author. The data are not publicly available due to ethical and privacy restrictions indicated in informed consent.
